# Predicting the effects of climate change on *Schistosoma mansoni* transmission in eastern Africa

**DOI:** 10.1186/s13071-014-0617-0

**Published:** 2015-01-06

**Authors:** Nicky McCreesh, Grigory Nikulin, Mark Booth

**Affiliations:** School of Medicine, Pharmacy and Health, Durham University, Durham, DH1 3LE UK; Swedish Meteorological and Hydrological Institute, Rossby Centre, Norrköping, SE-601 7 Sweden

**Keywords:** Schistosomiasis, *Biomphalaria*, Malacology, Climate change, Disease modelling, Africa

## Abstract

**Background:**

Survival and fitness attributes of free-living and sporocyst schistosome life-stages and their intermediate host snails are sensitive to water temperature. Climate change may alter the geographical distribution of schistosomiasis by affecting the suitability of freshwater bodies for hosting parasite and snail populations.

**Methods:**

We have developed an agent-based model of the temperature-sensitive stages of the *Schistosoma mansoni* and intermediate host snail lifecycles. The model was run using low, moderate and high warming climate projections over eastern Africa. For each climate projection, eight model scenarios were used to determine the sensitivity of predictions to different relationships between air and water temperature, and different snail mortality rates. Maps were produced showing predicted changes in risk as a result of increasing temperatures over the next 20 and 50 years.

**Results:**

Baseline model output compared to prevalence data indicates suitable temperatures are necessary but not sufficient for both *S. mansoni* transmission and high infection prevalences. All else being equal, infection risk may increase by up to 20% over most of eastern Africa over the next 20 and 50 years. Increases may be higher in Rwanda, Burundi, south-west Kenya and eastern Zambia, and *S. mansoni* may become newly endemic in some areas. Results for 20-year projections are robust to changes in simulated intermediate host snail habitat conditions. There is greater uncertainty about the effects of different habitats on changes in risk in 50 years’ time.

**Conclusions:**

Temperatures are likely to become suitable for increased *S. mansoni* transmission over much of eastern Africa. This may reduce the impact of control and elimination programmes. *S. mansoni* may also spread to new areas outside existing control programmes. We call for increased surveillance in areas defined as potentially suitable for emergent transmission.

**Electronic supplementary material:**

The online version of this article (doi:10.1186/s13071-014-0617-0) contains supplementary material, which is available to authorized users.

## Background

The transmission potential of most neglected tropical diseases in a particular location is partly dependent on abiotic factors affecting either free-living life stages and/or those which occur in poikilothermic organisms such as snails and mosquitoes. As an exemplar, both the schistosome parasite and its intermediate host snails are very sensitive to water temperature [1-4]. Increasing temperatures in freshwater bodies in sub-tropical and tropical areas may therefore alter the geographic distribution of schistosomiasis. There is some empirical evidence that this may be occurring already in Uganda, with transmission occurring at altitudes previously considered too cold [5,6].

Climate change projections show increasing temperatures across Africa [7], where the majority of people infected with schistosome parasites are located [8]. What remains unclear is how this phenomenon might affect the transmission potential of schistosomiasis in different locations, given the non-linear relationship between water temperature and schistosome transmission [9].

Perhaps because of the lack of research into this issue, the implications of climate change for schistosomiasis control and elimination have been largely ignored, and were not mentioned in the World Health Organization’s 2012 ‘Roadmap to implementation’ [8]. Only a few mathematical models have explored the effects of temperature on schistosomiasis transmission [9-15], and while some incorporated projected increases in mean temperature, no previous models have been run using climate projections. Although many geostatistical models of schistosomiasis prevalence incorporate temperature (e.g. [16-18]) (and, in one case, climate change [19]), limitations of the empirical data and the complex, non-linear relationship between water temperature and infection risk [9] mean that they are unlikely to capture the full contribution of temperature. In this study, we advance the field by producing, for the first time, high-resolution maps of eastern Africa highlighting areas where temperatures may become suitable for increased or decreased transmission, and where *Schistosoma mansoni* may spread to new areas.

## Methods

### Model

**Figure 1:**
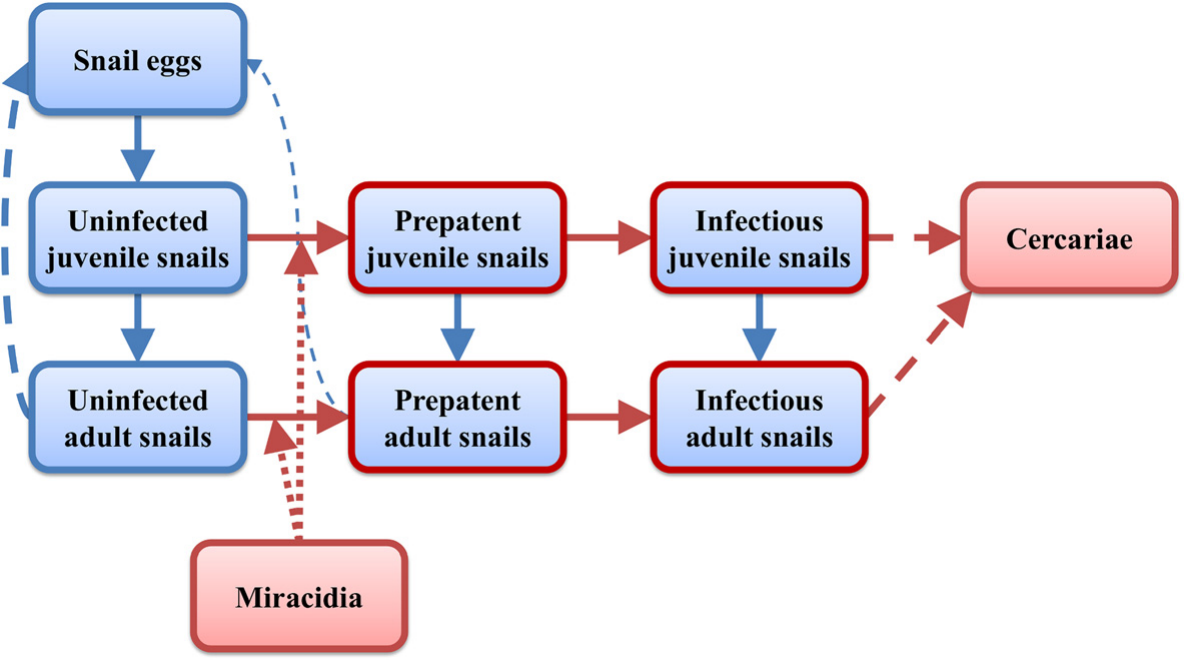
**Model structure.** Boxes show types of agents. Solid arrows show where agents can change from one type to another. Dashed lines show the production of one type of agent by another. Dotted lines indicate infection. Red outlines and arrows indicate the presence of schistosomes. Agents of all types can die and be removed from the model.

The model used in this study is described in full in McCreesh and Booth [9]. Briefly, the model is an agent-based model, written in Netlogo [20]. Snail eggs, juveniles and adults; prepatent and infectious snails; cercariae; and miracidia are represented as agents with temperature dependent birth or production, development and mortality rates. A diagram of the model structure is given in Figure 1. The model is parameterised using experimental and field data from *Biomphalaria pfeifferi*, the most widespread *S. mansoni* intermediate host snail species in sub-Saharan Africa [19], and the parasite.

The main output of the model is ‘infection risk’, a measure of the mean number of cercariae in the model each hour, adjusted for the fact that their probability of causing infection decreases as the amount of time since they were shed from the snail increases. It can be viewed as an estimate of the suitability of temperatures for schistosome transmission in an area. The non-temperature dependent stages of the schistosome lifecycle – the stages in humans – are not simulated, and the rate of miracidium introduction into the model is constant.

The model has a time step of one hour, allowing the cercariae and miracidia, which have lifespans of the order of hours, to be accurately represented. Hourly temperatures are simulated using a sine wave, with maximum temperatures reached at 3 to 4 pm, and minimum temperatures at 3 to 4 am [21].

To allow schistosomes to become established in the model in new locations, a snail egg is introduced into the model each hour with a probability of 0.00012 (which gives an average rate of one snail egg per model year). As non-temperature dependent egg mortality is simulated using reduced egg production rates in the model, this is equivalent to a ‘real life’ egg introduction rate of 10 eggs per year.

### Climate projections

An ensemble of regional climate simulations over Africa was used to provide projected daily maximum and minimum temperature data for the eastern Africa study region. The ensemble consists of three members of the Rossby Centre Regional Climate Model (RCM) – RCA4 [22], driven by a coupled atmosphere ocean general circulation model (AOGCM) – EC-EARTH [23]. Three Representative Concentration Pathway (RCP) scenarios were used – RCP2.6, RCP4.5 and RCP8.5 – which represent low, moderate and high levels of warming respectively. All three regional simulations were made within the African branch of the Coordinated Regional Downscaling Experiment (CORDEX), and cover the whole African continent at about a 50 km (0.44°) resolution. A smaller sub-domain in eastern Africa was selected for the study and has size of 1470 (35 x 42) grid boxes.

The mean absolute increases in temperature over the study area over the next 20 years (2026–2035 relative to 2006–2015) were 0.34°C, 0.42°C, and 0.66°C in the low, moderate, and high warming projections, respectively. Over the next 50 years (2056–2065 relative to 2006–2035) the mean increases were 0.53°C, 1.2°C, and 1.8°C (Additional file 1: Figure S1). To prevent confusion, the different climate projections or scenarios are referred to as ‘projections’ throughout the paper, and the term ‘scenarios’ reserved for the model scenarios described below.

### Model scenarios

For each set of climate projections, the model was run for eight model scenarios. Four different relationships between air temperature and water temperature were simulated:

(i) daily minimum and maximum water temperatures equal to daily minimum and maximum air temperatures;
(ii) minimum and maximum water temperatures 2°C higher;
(iii) minimum and maximum water temperatures 2°C lower; and
(iv) minimum water temperatures 2°C higher, and maximum water temperatures 2°C lower.

These were each simulated using two sets of snail mortality rates: rates estimated from experimental and field data [9], and double these rates. Therefore, in total, eight model scenarios were simulated for each of three climate projections.

The model was initially run using the first two years of the climate data (2006–2008) to allow snail numbers to reach plausible initial values. Following this, the model was run using the full climate data between 2006 and 2065. The model was run separately for each of the 1470 grid squares. For each location, the model was run 20 times and the results averaged. Outputs were averaged over each of three 10-year time periods: baseline (2006–2015), 20 years in the future (2026–2035), and 50 years in the future (2056–2065). Kernel interpolation using a fifth order polynomial function was used to produce smooth risk maps for each scenario and time period.

### Comparison with empirical data

Geo-referenced data on the prevalence of *S. mansoni* in human populations in the model output region were extracted from the open-access Global Neglected Tropical Disease (GNTD) database [24]. The database brings together historical and contemporary survey data on schistosomiasis and other neglected tropical diseases, and the data have been widely used in statistical models of disease prevalence (e.g. [18,19]). For grid squares containing more than one data point, the un-weighted mean of the prevalence estimates was calculated. The mean prevalence for each grid square was then plotted against the mean (across scenarios) model output ‘infection risk’ for the same square, and the area under the receiver operating characteristic curve (AUC) calculated for the ability of the model to predict prevalences of above 0%, 10%, 20% and 50%. The analysis was then repeated, restricting the prevalence data to estimates from children, collected from 2004 onwards, and where at least 10 children were tested (‘selected data’).

### Analysis

To enable easy interpretation of model results, for each climate projection and future time period median changes in risk (across model scenarios) were calculated to give a central estimate of the magnitude of changes that may occur. Means were not used as the predicted changes in each scenario were highly skewed in some locations, due to very high relative risks in some areas and scenarios where baseline risks were very low.

The level of agreement between scenarios in the direction of change in risk was also investigated. For each scenario, climate projection, and future time period, areas were given the value of +1 if risk was predicted to increase from its baseline value, −1 if risk was predicted to decrease, and zero if it was predicted to stay the same. Risk was considered to stay the same if it changed by less than ±10%. The values were then summed over all eight scenarios for each climate projection and future time period to give an indication of the overall predicted direction of change. For areas where both an increase and a decrease in risk were predicted by one or more scenarios, the number of scenarios that disagreed with the overall predicted direction of change was calculated.

Additional analyses were conducted on each of the model scenarios to explore the possibility of schistosome transmission becoming established at new sites. For these analyses, areas were given the value of +1 if risk was predicted to increase from below to above a cut-off between the two time periods, and zero otherwise. The analysis was conducted for all possible cut-offs between 1% and 99% of the maximum risk in the scenario in any time period, increasing in increments of 1% of the maximum risk. The cut-offs were split into three equal-sized groups (1-33%, 34-66%, and 67-99% of maximum risk), and the proportion of cut-offs crossed was calculated for each location, for each group, time period and climate projection.

## Results

### Comparison with empirical data

**Figure 2:**
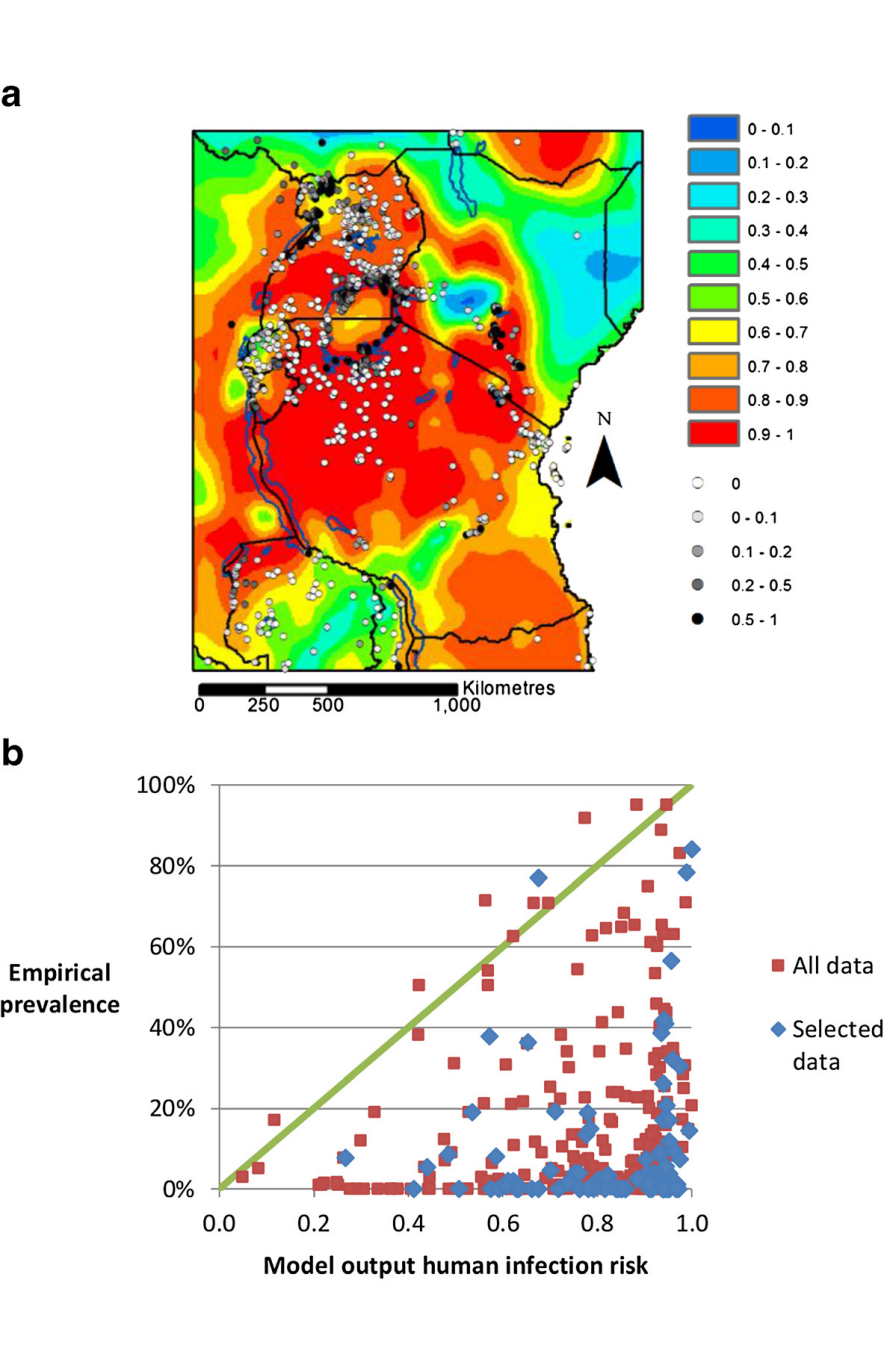
**Comparison of model output ‘infection risk’ at baseline (2006–2015) with empirical prevalence estimates. a)** Baseline risk map. Background colour shows model output ‘infection risk’, averaged across scenarios, and translated into proportion of maximum risk. Greyscale circles show empirical prevalence data. **b)** Model output ‘infection risk’ plotted against empirical prevalence data. The red squares show mean prevalence for all 279 grid squares for which any prevalence data were available. The blue diamonds show mean prevalence for 100 grid squares for which higher quality and more suitable prevalence data were available (‘selected data’). Infection risk has been translated into proportion of maximum infection risk. The green line shows where prevalence and translated infection risk are equal.

Empirical prevalence data were available from 2965 records in total, and 594 records when surveys that did not meet stricter inclusion criteria were excluded (‘selected data’). Figure 2a illustrates prevalence data plotted on a map of the mean (across scenarios) model output ‘infection risk’ at baseline (2006–2015). The prevalence data yielded estimates of prevalence for 19% (279/1470) of grid squares when all data were used, and 7% (100/1470) when selected data were used (Table 1). Prevalence estimates for each square were calculated from 1–119 (median = 4) and 1–37 (median = 4) individual estimates when all and selected data were used respectively.

**Table 1:** **Comparison of model output ‘infection risk’ at baseline (2006–2015) with empirical prevalence estimates**

	**All data (N = 279)**		**Selected data (N = 100)**	
**Prevalence cut-off**	**Number above cut-off (%)**	AUC* (95% CI)	**Number above cut-off (%)**	AUC* (95% CI)
0%	209 (75)	0.60 (0.52-0.67)	66 (66)	0.56 (0.45-0.68)
10%	98 (35)	0.62 (0.55-0.69)	22 (22)	0.62 (0.47-0.77)
20%	69 (25)	0.63 (0.56-0.71)	13 (13)	0.68 (0.50-0.86)
50%	26 (9)	0.57 (0.46-0.69)	4 (4)	0.76 (0.39-1.00)

*Area under receiver operating characteristic curve.

When all data were used, the AUC’s were 0.6 (0.52-0.67), 0.62 (0.55-0.69), 0.63 (0.56-0.71), and 0.57 (0.46-0.69) for prevalence cut-offs of above 0%, 10%, 20% and 50% respectively. When selected data were used, the AUC’s were 0.56 (0.45-0.68), 0.62 (0.47-0.77), 0.68 (0.50-0.86), and 0.76 (0.39-1.00) (Table 1). Figure 2b illustrates the mean model output at baseline divided by the maximum model output at any location, plotted against the prevalence data. The mean prevalence is higher than the adjusted model output in 9/279 (3.2%) grid squares when all data were used, and 1/100 (1.0%) grid squares when selected data were used.

### Increase in risk

**Figure 3:**
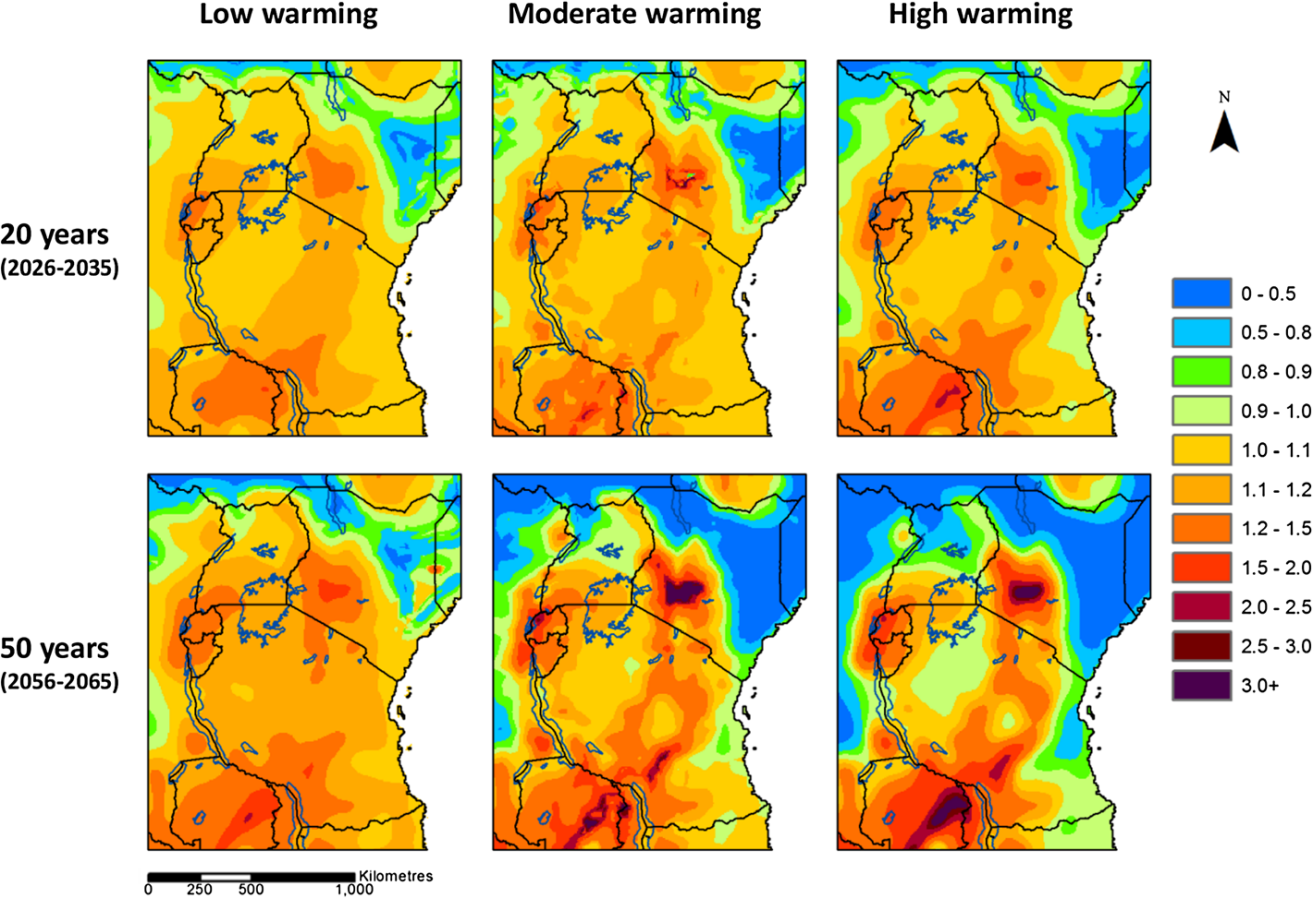
**Median predicted change in** ***S. mansoni*** **risk in eastern Africa.** Results are for 2026–2035 relative to 2006–2015 (top) and 2056–2065 relative to 2006–2015 (bottom). Median is calculated across eight model scenarios for each map.

**Figure 4:**
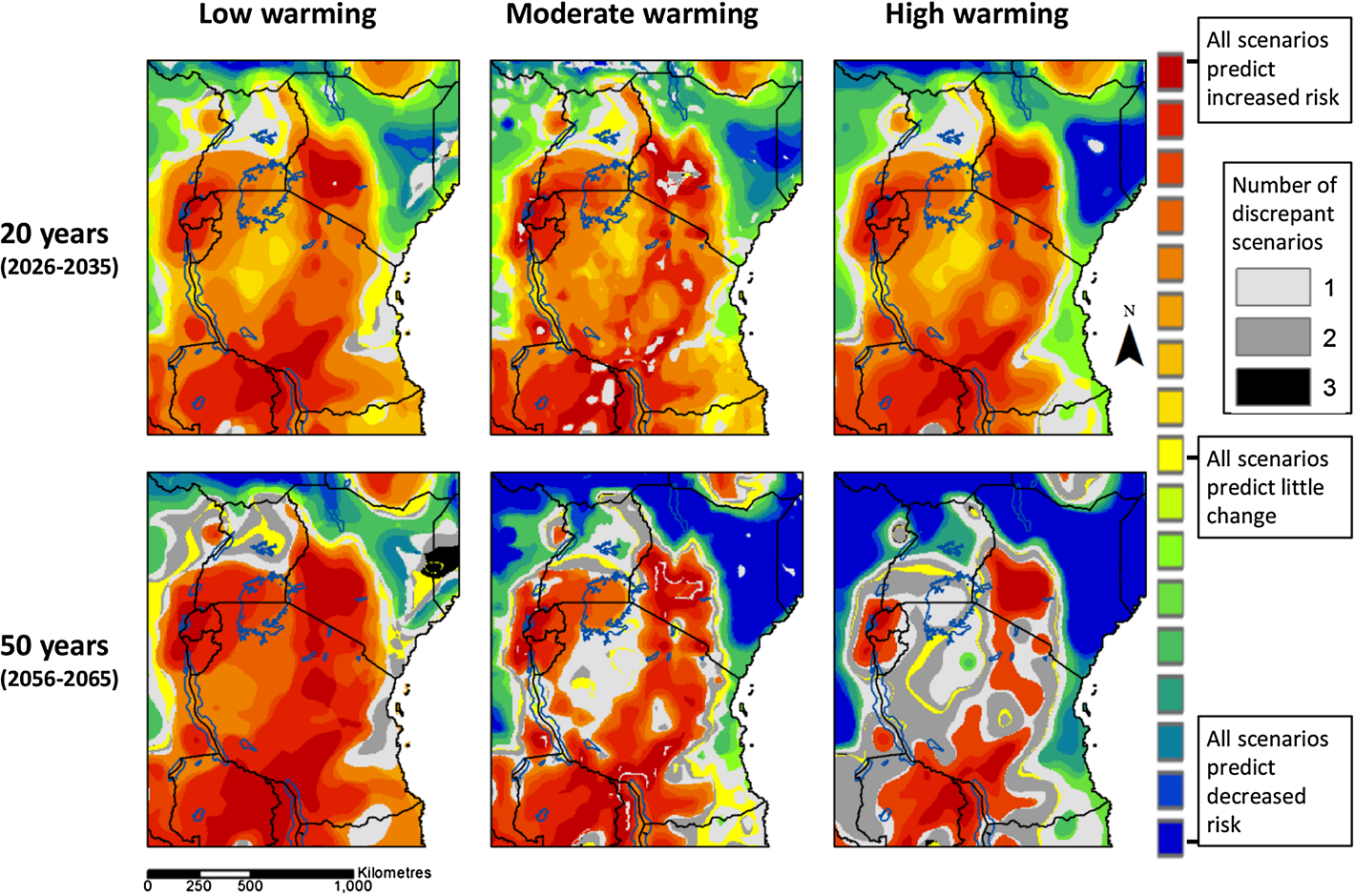
**Agreement between scenarios in the direction of change in** ***S. mansoni*** **risk in eastern Africa.** Results are for 2026–2035 relative to 2006–2015 (top) and 2056–2065 relative to 2006–2015 (bottom). Areas are shown in yellow if all scenarios agree that increasing temperatures will have little effect on schistosomiasis transmission. Areas are shown in red and blue respectively if there is widespread agreement between scenarios that temperatures will become suitable for increased or decreased schistosomiasis transmission over the next 20 years. Areas are shown in grey if the majority of scenarios predict increasing risk or little change, but one or more scenarios predict decreasing risk, or *vice versa*.

Figure 3 illustrates the median predicted change in *S. mansoni* infection risk across scenarios, over the next 20 and 50 years. Figure 4 gives an indication of the level of agreement between scenarios in the overall direction of change (increased risk or decreased risk), and the number of scenarios that disagree with the overall direction. There is widespread agreement between scenarios and climate projections that infection risk may increase in Rwanda, Burundi, and eastern Zambia and over most of Uganda, Tanzania and south-west Kenya over the next 20 years, and that infection risk may decrease in north-east Kenya. A similar picture is found in 50 years’ time, with the exception of the high warming scenario where risk is predicted to decrease over larger areas, and where there is disagreement between scenarios in the direction of change in risk over larger areas. In the majority of areas, the median predicted increase in infection risk is less than 20%. In parts of Rwanda, Burundi, south-west Kenya and eastern Zambia, however, the median increase in risk is higher.

There is widespread agreement between scenarios that infection risk may decrease by more than 50% over the next 20 and 50 years in parts of north and east Kenya, southern South Sudan, and eastern People’s Democratic Republic of Congo. The size of the area over which reductions may occur is larger with higher levels of warming, and in 50 years’ time.

### Newly endemic areas and new foci of transmission

**Figure 5:**
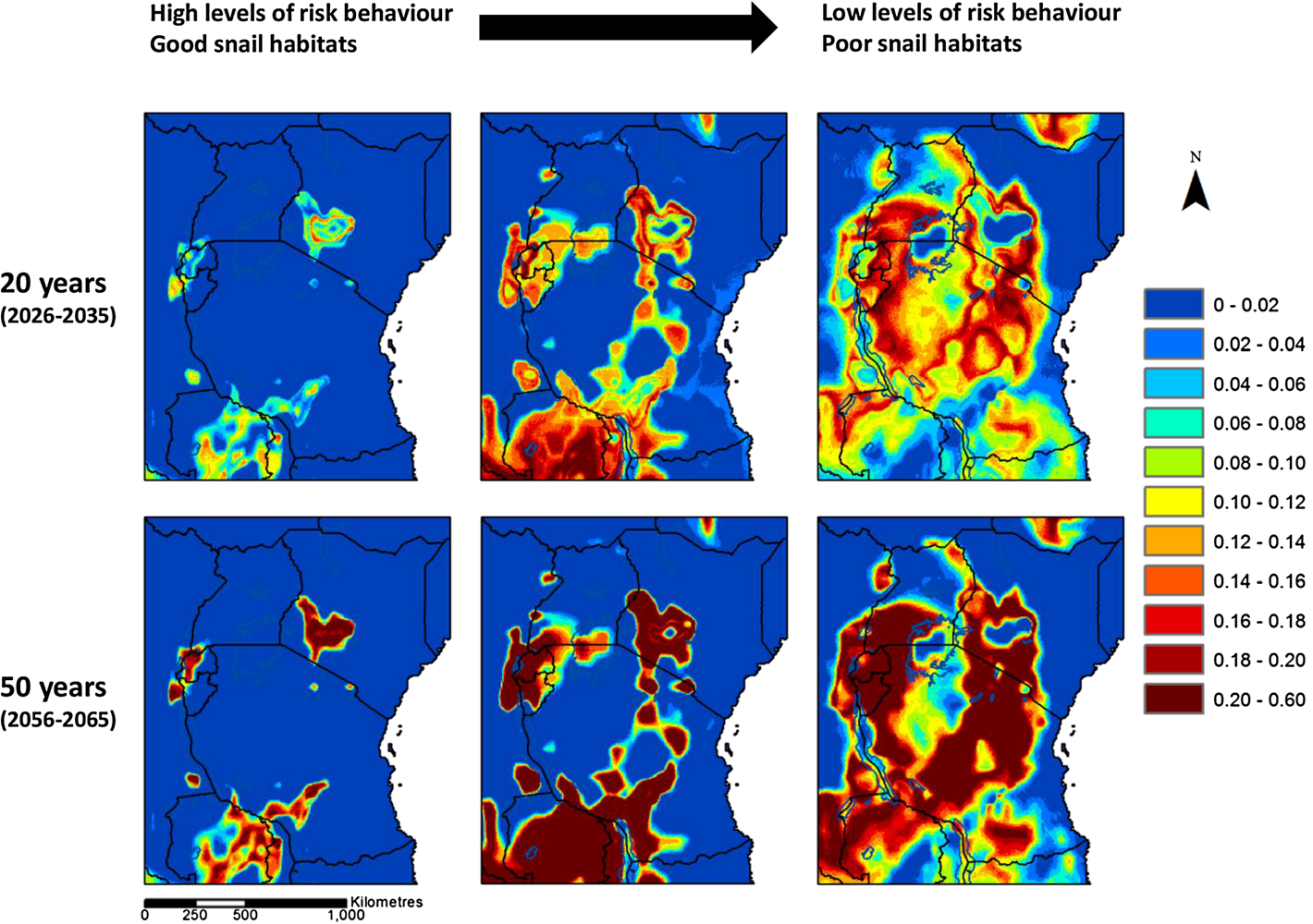
**Relative risk of new foci of transmission developing, using the moderate warming climate projection.** Results are for 2026–2035 relative to 2006–2015 (top) and 2056–2065 relative to 2006–2015 (bottom). Blue colours indicate little or no risk. Red colours indicate high risk. The maps on the left show risk in villages with high levels of risk behaviour and good snail habitats. The maps on the right show risk in villages with lower levels of risk behaviour and/or poor snail habitats. The key indicates the proportion of cut-offs that were crossed between baseline and 20 and 50 years’ time. Results for the low and high warming scenarios are shown in the Additional file 2: Figures S2 and Additional file 3: Figure S3.

Figure 5 highlights areas at risk of new transmission foci developing. The left-hand maps show areas where the model predicts that cut-offs corresponding to 1-33% of maximum risk will be crossed over the next 20 and 50 years. These cut-offs correspond to temperatures which are suitable for transmission, but not ideal. These cut-offs are therefore most likely to be crossed in areas where both levels of human risk behaviour are high, and where highly suitable snail habitats are found (for instance permanent habitats with a good supply of food and few predators). The right-hand maps illustrate areas where the model predicts that cut-offs corresponding to 67-99% of maximum risk will be crossed. These cut-offs correspond to temperatures which are highly suitable for transmission. Schistosome transmission may therefore newly occur in villages and at potential transmission sites where levels of human risk behaviour are lower and/or snail habitats are more marginal. Figure 5 shows the results for the moderate warming projections only. The results for the low and high warming projections are similar and are shown in Additional file 1: Figures S2 and Additional file 2: Figure S3.

## Discussion

The results suggest that, all else being equal, *S. mansoni* infection risk may increase across much of eastern Africa as temperatures increase over the next few decades. In most areas, the predicted increases are less than 20%. Temperature driven increases in risk may be much larger in Rwanda, Burundi, south-west Kenya, and eastern Zambia. Conversely, infection risk may decrease by more than 50% in parts of north and east Kenya, southern South Sudan, and eastern People’s Democratic Republic of Congo. The results also highlight areas where schistosome transmission may occur at new sites.

The results predict changes in risk that are attributable to increasing temperatures only. Other climatic changes, such as changes in patterns of rainfall, flooding and droughts, will also have an impact on future schistosomiasis prevalence [25]. Changes in patterns of rainfall may be particularly important in determining the seasonality of transmission in many areas, and therefore results are not presented by season. In addition to climatic changes, non-climatic changes will play a large role in determining future prevalence and infection intensity. These include changes in land use, population growth and mobility, water contact behaviour and sanitation infrastructure. Potential modifying factors of risk also include mass treatment control programmes, and snail control efforts. For these reasons, the model results should not be taken as predictive of future schistosomiasis prevalence as such, but instead as indicative of areas where temperature changes are likely to influence schistosomiasis transmission potential.

A novel feature of the model presented here is consideration of the relationship between air and water temperature in different types of water body and in different seasons. Compared to minimum and maximum air temperatures, minimum and maximum water temperatures may be both higher [21,26], both lower [26], or may be higher and lower respectively [26,27]. Furthermore, snails may be capable of escaping above optimum water temperatures by moving to deeper water [25], where water temperatures may be up to 2°C cooler [28], or by burrowing in mud [25]. For these reasons, a number of scenarios were simulated, with different relationships between air temperature and water temperature. Scenarios with high and low snail mortality rates explored the effect of differences between different types of habitat further. With the exception of the moderate and high warming projections in 50 years’ time, there is little disagreement between scenarios in the direction of change in risk, suggesting that the model results are robust to variations between snail habitats. The eight scenarios also act as a form of sensitivity analysis, as shifting temperatures and keeping snail and parasite temperature preferences constant is equivalent to keep the temperatures constant but altering model parameterisation.

Validation of the model is challenging as the model produces an estimate of the contribution of temperature to infection risk, whereas infection prevalence is the standard measure of population infection risk. Estimating prevalence was outside of the scope of this study due to a lack of available information (at high spatial resolution) on most of the underlying processes and phenomena that drive infection prevalence, including the presence or absence of suitable snail habitats, water contact behaviour, and sanitation infrastructure and usage. In addition, prevalence is a poor indicator of infection risk, and data on infection intensity are rarely routinely collected [29]. Existing prevalence data are also of varying quality, having been collected for a wide range of different purposes, at different times, in different age groups, using different sampling methods, and using different tests with widely varying sensitivities [30]. Areas where temperatures are considered to be unsuitable for schistosomiasis may be greatly under-sampled, and these are the areas where the predictive ability of the model is likely to be highest. These limitations both impede model validation, and reduce the ability of geostatistical models to accurately capture the effects of temperature.

Despite the limitations of the empirical data, a comparison of model output at baseline with prevalence data gives AUC’s of around 0.6 (Table 1), which suggests that temperature does play an important role in determining empirical prevalence (and that the model is able to capture that role), but that other factors are also important. Figure 2 illustrates clearly that zero or low prevalences of schistosomiasis can occur in any area, regardless of temperatures, but that higher prevalences only occur in areas where temperatures are suitable for higher levels of transmission. In other words, suitable temperatures are necessary but not sufficient for both schistosome transmission and for high prevalences of schistosomiasis.

Snail eggs are introduced into the model at an average rate of one a year. This is necessary to allow simulated snail populations to become established in areas where conditions become newly suitable. In real snail populations, this may occur through movement of snails from interconnected water bodies or during flooding [31], or through live snails being transported short distances on objects such as fishing nets. These events are all extremely stochastic, and the ‘newly endemic’ model results should therefore be taken to indicate areas that may become newly suitable for the establishment of schistosome transmission only, and not as a definite guide to the spread of schistosomiasis. The exception to this is cooler areas where snails are found but schistosome transmission does not currently occur, where new transmission foci could quickly become established as temperatures become suitable.

Taking regional climate simulations made by only one regional climate model, downscaling one global circulation model (under different RCP scenarios), is not enough to assess the full range of uncertainties in future climate projections. However, the main focus of the study is to provide a range of plausible future changes in schistosomiasis risk, suitable for public health planning purposes, and to explore the effect of uncertainties associated with the disease model and its parameterisation. Using a wider range of different climate projections (from different RCMs downscaling different AOGCMs) is unlikely to have any real effect on the public health consequences of our findings. As the main focus of the study was infectious disease modelling, there was also no bias correction applied to the RCM data.

To maximise the potential utility of our results for public health purposes, we used short (10-year) time slices for the analysis, namely: 2006–2015 (baseline), 2026–2035 and 2056–2065. Using 10-year time periods can lead to errors if natural multi-decadal variability is attributed to the global warming signal. We do not believe that this was the case for our study however, as climate projections for daily maximum and minimum temperatures in eastern Africa averaged over 10-years were robust and consistent across the three RCP trajectories (Additional file 1: Figure S1).

The model was parameterised using data from *B. pfeifferi* snails. This is the most widespread intermediate host snail species in sub-Saharan Africa [19]. Other species of intermediate host snail are found at many transmission sites however, and previous modelling work has shown that the species of snail used in parameterising a model can have a large effect on the relationship between water temperature and infection risk [15]. Simulating different relationships between air temperature and water temperature goes some way towards testing the sensitivity of the model to the choice of snail species, as the higher and lower simulated temperatures could also be viewed as alterations in minimum, maximum, and optimum temperatures for snail survival and reproduction. The widespread agreement between scenarios therefore suggests that the model is not very sensitive to small differences in temperature preferences between snail species. Nevertheless, experimental work with the different species of intermediate host snail found in eastern Africa would increase confidence in the model results by allowing the model to be explicitly parameterised to other species of snail.

A practical application of the model presented here is associated with planning the long-term control of schistosomiasis. Current efforts rely on periodic mass administration of medicines within specific areas. As climate change begins to exert an influence on the African environment, the situation on the ground is likely to change. Mitigation and/or adaptation measures to climate change will require greater surveillance efforts to capture those changes in advance of them becoming significantly deleterious. A rapid mapping of snail populations within the areas identified as being at risk of new endemicity would pinpoint the communities at highest risk, allowing targeted monitoring of human populations.

## Conclusions

Climate change may have a large effect on both the distribution and intensity of *S. mansoni* infection over coming decades. Temperatures are predicted to become suitable for increased transmission over much of eastern Africa over the next 20 years. This may lead to increased prevalences and intensities of infection in some areas, and is likely to reduce the effects of control and elimination programmes. In some areas, particularly in Rwanda, Burundi, south-west Kenya and eastern Zambia, increases in infection risk may be large. Schistosomiasis may spread to new areas, outside the current range of control programmes. Increased surveillance of these areas would enable education and control programmes to be promptly implemented in newly endemic areas, minimising disease morbidity.

## Additional files

Additional file 1: Figure S1. Projected changes (relative to 2006-2015) in maximum and minimum temperatures under RCP2.6, RCP4.5 and RCP8.5, averaged over the eastern Africa study region and smoothed using a 10-year moving average. Vertical lines mark 2006-2015, 2026-2035 and 2056-2065. The numbers show the increase in temperature (°C). Additional file 2: Figure S2. Relative risk of new foci of transmission developing between 2006-2015 and 2026-2035 (top) and 2056-2065 (bottom), using the low warming climate projection. Blue colours indicate little or no risk. Red colours indicate high risk. The maps on the left show risk in villages with high levels of risk behaviour and good snail habitats. The maps on the right show risk in villages with lower levels of risk behaviour and/or poor snail habitats. The key indicates the proportion of cut-offs that were crossed between baseline and 20 and 50 years’ time. Additional file 3: Figure S3. Relative risk of new foci of transmission developing between 2006-2015 and 2026-2035 (top) and 2056-2065 (bottom), using the high warming climate projection. Blue colours indicate little or no risk. Red colours indicate high risk. The maps on the left show risk in villages with high levels of risk behaviour and good snail habitats. The maps on the right show risk in villages with lower levels of risk behaviour and/or poor snail habitats. The key indicates the proportion of cut-offs that were crossed between baseline and 20 and 50 years’ time.
