# Supplementary material for: Predicting the effects of climate change on Schistosoma mansoni transmission in eastern Africa
Source: Parasit Vectors. 2015 Jan 6;8:4. doi: 10.1186/s13071-014-0617-0 (PMC4297451; doi:10.1186/s13071-014-0617-0)
Supplement: Additional file 1: Figure S1. — Projected changes (relative to 2006-2015) in maximum and minimum temperatures under RCP2.6, RCP4.5 and RCP8.5, averaged over the eastern Africa study region and smoothed using a 10-year moving average. Vertical lines mark 2006-2015, 2026-2035 and 2056-2065. The numbers show the increase in temperature (°C). [file 13071_2014_617_MOESM1_ESM.docx]

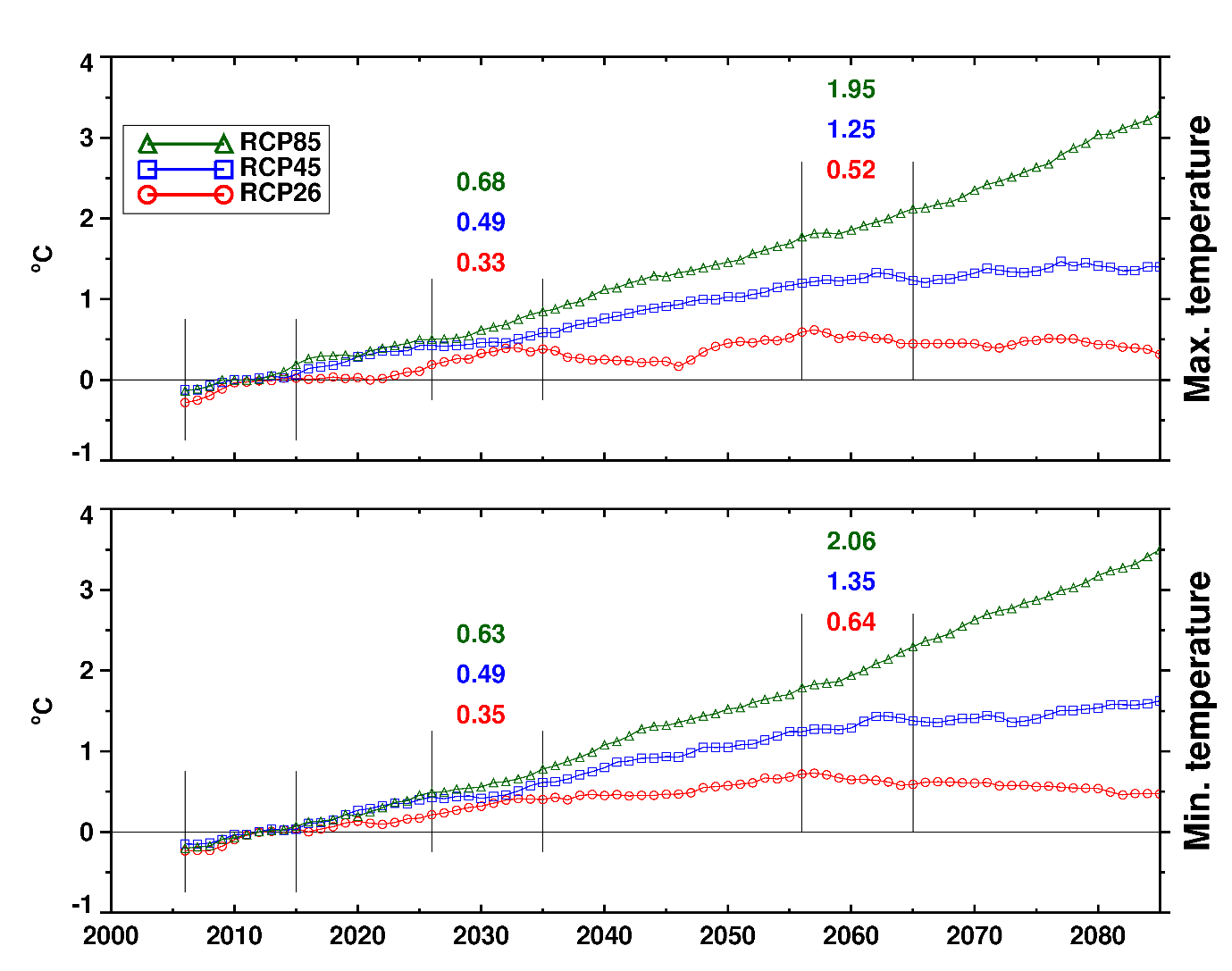


**Supplementary figure S1. Projected changes (relative to 2006-2015) in maximum and minimum temperatures under RCP2.6, RCP4.5 and RCP8.5, averaged over the eastern Africa study region and smoothed using a 10-year moving average**. Vertical lines mark 2006-2015, 2026-2035 and 2056-2065. The numbers show the increase in temperature (°C).
