# Supplementary material for: Predicting the effects of climate change on Schistosoma mansoni transmission in eastern Africa
Source: Parasit Vectors. 2015 Jan 6;8:4. doi: 10.1186/s13071-014-0617-0 (PMC4297451; doi:10.1186/s13071-014-0617-0)
Supplement: Additional file 3: Figure S3. — Relative risk of new foci of transmission developing between 2006-2015 and 2026-2035 (top) and 2056-2065 (bottom), using the high warming climate projection. Blue colours indicate little or no risk. Red colours indicate high risk. The maps on the left show risk in villages with high levels of risk behaviour and good snail habitats. The maps on the right show risk in villages with lower levels of risk behaviour and/or poor snail habitats. The key indicates the proportion of cut-offs that were crossed between baseline and 20 and 50 years’ time. [file 13071_2014_617_MOESM3_ESM.docx]

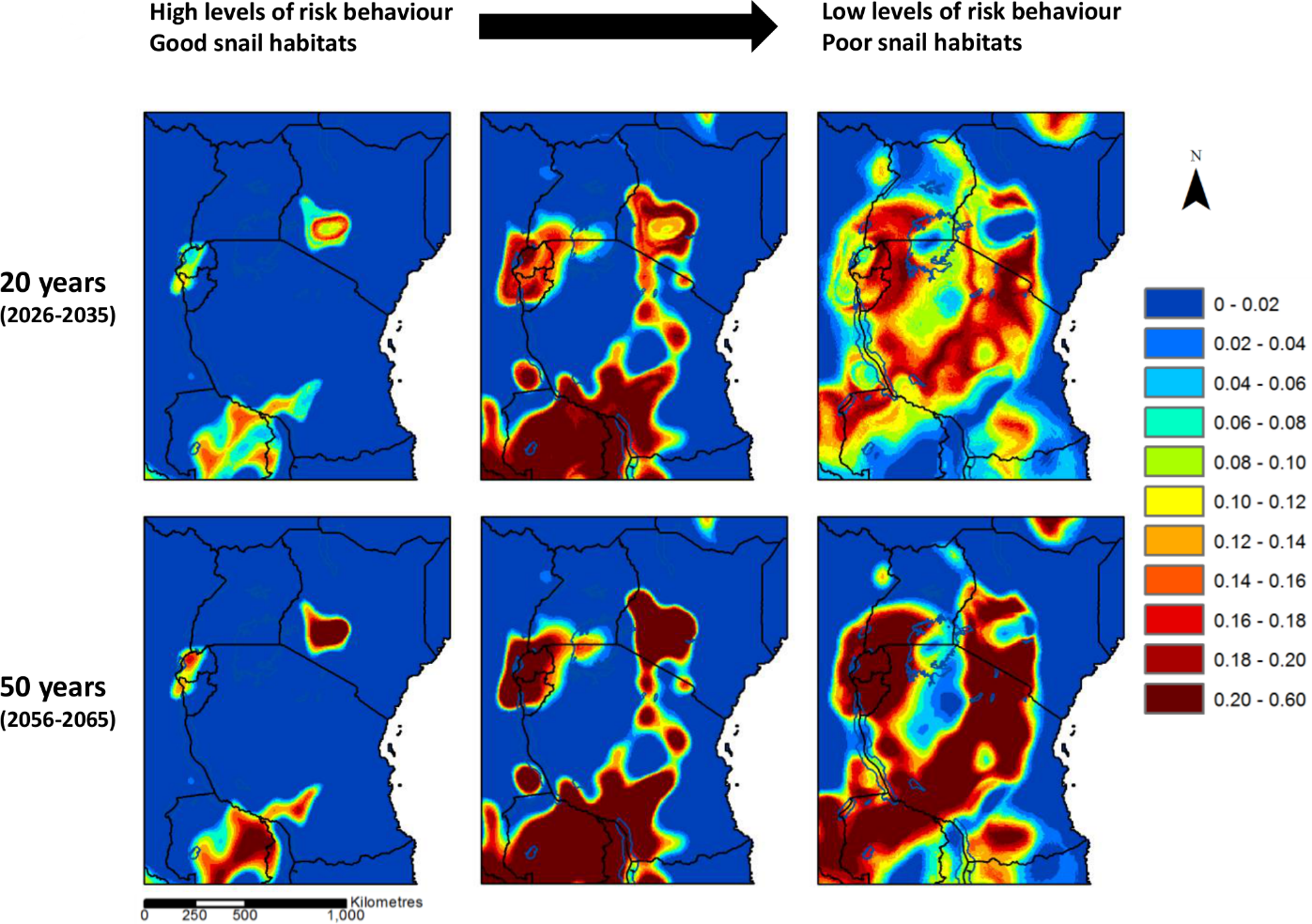


**Supplementary figure S3. Relative risk of new foci of transmission developing between 2006-2015 and 2026-2035 (top) and 2056-2065 (bottom), using the high warming climate projection.** Blue colours indicate little or no risk. Red colours indicate high risk. The maps on the left show risk in villages with high levels of risk behaviour and good snail habitats. The maps on the right show risk in villages with lower levels of risk behaviour and/or poor snail habitats. The key indicates the proportion of cut-offs that were crossed between baseline and 20 and 50 years’ time.
